# Surgical Helmets and SARS Infection

**DOI:** 10.3201/eid1002.030764

**Published:** 2004-02

**Authors:** James L. Derrick, Charles D. Gomersall

**Affiliations:** * The Chinese University of Hong Kong, Shatin, Hong Kong Special Administrative Region, People’s Republic of China

**Keywords:** Respiratory protective devices, severe acute respiratory syndrome, safety, occupational, disease transmission, patient-to-professional, aerosols, infection control

## Abstract

Performance testing of two brands of surgical helmets indicated that their efficiency at in vivo filtration of sub–micrometer-sized particles is inadequate for their use as respirators. These helmets are not marketed for respiratory protection and should not be used alone for protection against severe acute respiratory syndrome when performing aerosol-generating procedures.

Severe acute respiratory syndrome (SARS) is a highly contagious, potentially life-threatening condition that frequently affects healthcare workers caring for infected patients (*1*). Healthcare workers may need to adopt additional infection control procedures when carrying out potentially high-risk procedures such as intubation and surgery (*2*). These procedures can generate aerosols known to penetrate surgical masks (*4*,*5*), which may contaminate all staff in the operating room (*3*). Furthermore, other viruses such as the human papillomavirus have been shown to be present in CO_2_ laser and diathermy plumes (*6*,*7*).

Surgical helmets such as the Stryker T4 (Stryker Instruments, Kalamazoo, MI) and Stackhouse FreedomAire (Stackhouse Incorporated, Palm Springs, CA) cover the entire head and use a head-mounted fan to circulate air. Unlike powered air-purifying respirators (PAPRs), which draw ambient air through a HEPA filter and blow it over the face at such a high flow rate that no unfiltered air is entrained during inspiration, surgical helmets filter air through the hood material itself. In laboratory testing, the hood material of the Stryker filters 98% of 0.1-μm particles, according to Stryker Instruments. The Stackhouse helmet has an additional filter in front of the fan, which improves the filtering capacity for 0.12-μm particles to 99.6%, according to its manufacturer.

These devices are intended to decrease contamination of the surgical wound and to protect staff from splashes of bloodborne pathogens. Although these devices are not marketed as respirators, it is natural to consider that they may be helpful in preventing respiratory transmission of SARS. The efficiency of the helmets in decreasing bacterial contamination has been tested (*8*); however, how well these devices protect the wearer from airborne contaminants is not known.

## Materials and Methods

We carried out a prospective, unblinded study in six healthy volunteers at the Prince of Wales Hospital in Shatin, Hong Kong. We compared the filtration capacity of the Stryker T4 and Stackhouse FreedomAire surgical helmets with an 8233 N100 filtering facepiece respirator (3M, St. Paul, MN) combined with a surgical mask and full face shield. All volunteers gave written informed consent. Approval was obtained from the Clinical Research Ethics Committee of the Chinese University of Hong Kong.

Each participant performed one test with each device. Each test measured the ability of the device to filter ambient dust particles, normally present in room air, by using a previously described standard, quantitative, fit-testing protocol (*9*). In brief, the testing compared particle counts inside and outside the protective device during a series of activities—normal breathing, deep breathing, turning the head from side to side, flexing and extending the head, talking loudly, and bending over followed by normal breathing.

The tube for sampling the mask particle count was connected to a test probe designed for this purpose (TSI Incorporated, St. Paul, MN), which was inserted through the fabric of the protective device. On the N100 respirator, the probe was passed through both the respirator and covering surgical mask 1cm to the right of the valve. On the surgical helmets, the probe was placed centrally in the breathing zone 1 cm below the bottom edge of the transparent face piece. The tube for sampling the ambient particle count was fixed approximately 3 cm from the sampling probe. No participant had been previously fit tested on this brand of N100 respirator; however, all participants received instructions on donning both the respirators and the surgical helmets before use. Before each test we checked that all participants were wearing their devices correctly.

A PortaCount Plus (TSI Incorporated) connected to a computer running FitPlus for Windows software (TSI Incorporated) was used to count particles and calculate the ratio of ambient-to-device particle counts. This device counts all particles between 0.02 and 1 μm in diameter; it also calculates a fit factor, which is the average ratio of ambient-to-device particle concentrations. The equation used is 
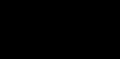


where: n is the number of exercises performed and ff_j_ is the fit factor for the individual exercise.

One modification was made to the PortaCount Plus. The reusable tubing supplied by the manufacturer was replaced with disposable polyvinyl chloride (PVC) tubing of the same internal diameter and length to minimize any risk for cross-infection. To ensure an adequate ambient particle count, the 8026 Particle Generator (TSI Incorported) was used to generate saline particles throughout the testing procedures. New hoods and masks were used for each participant. When the surgical helmet-hood combinations were being tested, the helmet and hood were put on and then a disposable surgical gown (MicroCool Specialty Gown, Kimberly-Clark, Roswell, GA) was worn over the top of the hood, in accordance with the manufacturer’s instructions. Since buildup of carbon dioxide has been found to be a problem with these helmets (*10*), the highest fan speed was used throughout the testing. During testing of the N100 mask, the participants wore a standard three-ply surgical mask (Surgicos Johnson & Johnson, Arlington, TX) tied over the top (since the N100 mask is not licensed for use as a surgical mask) and a full face shield (Splash Shield, Woburn, MA).

The median ratios of ambient-to-device particle counts were compared by using the Mann-Whitney U test (Statview 5.0, SAS Institute, Cary, NC). A p value <0.05 was considered significant.

## Results

During the tests, the median ambient concentration of 0.02- to 1-μm particles was 7,650/cm^3^ (range 3,980–29,200/cm^3^). Results of the filtration capacity of the three devices are shown in the Table. In all tests, the N100 mask filtered significantly more particles than either of the surgical helmet-hoods. During testing, a half-face respirator, such as the N100 mask, should reduce the particle count by a minimum of a factor of 100 (*11*). This minimum standard was exceeded with the N100 mask for all participants. The greatest particle count reduction achieved with a surgical helmet-hood was a factor of 4.8.

**Table:** Ratio of ambient-to-device concentrations of 0.02- to 1-μm–diameter particles (median [range])^a^

Exercise	Stryker T4	Stackhouse FreedomAire	3M 8233 N100 mask with surgical mask and face shield
Normal breathing	4.5 (4–5)	3 (2–4)	32,550 (1,420–60,900)
Deep breathing	4.5 (4–5)	3 (2–3)	21,550 (4,150–99,300)
Head side to side	4 (4–5)	3 (2–3)	15,675 (681–138,000)
Head up and down	4 (3–5)	3 (2–3)	19,300 (380–138,000)
Talking	4 (3–5)	3 (2–3)	1,550 (394–18,200)
Bending over	3.5 (3–4)	2 (2–3)	7,695 (1,620–31,000)
Normal breathing	4 (3–5)	2.5 (2–3)	22,100 (4,670–163,000)
Fit factor	3.8 (3.7–4.8)	2.5 (2.0–3.1)	6,392 (962–50,519)

^a^Ratios for Stryker T4 and Stackhouse FreedomAire were significantly lower in all tests compared to the combination of N100 mask, surgical mask, and face shield (p <0.004.)

## Discussion

Our data demonstrate that both surgical helmet-hoods have markedly inferior in vivo filtration performance compared to the combination of N100 mask, surgical mask, and face shield. More importantly, both surgical helmet-hoods failed in all cases to meet the National Institute for Occupational Safety and Health performance requirement for even a half-mask respirator. The requirement for a PAPR is higher. Clearly, this failure rate would be unacceptable if these devices were to be considered for use as respirators. Neither surgical helmet is approved as a respirator nor marketed as a method of protecting the user against respiratory pathogens. In fact, Stryker recommends that its helmet be used in combination with additional eye and respiratory protection in this setting (available from: URL: http://sars.medtau.org/strykerreport.doc).

Several caveats need to be applied when interpreting our data. First, we tested filtration of particles, not the coronavirus postulated to cause SARS. In addition, it is impossible to be certain what size of particles the surgical helmet-hoods were failing to adequately filter, nor is it obvious which particle size is most important to filter, since many aerosolized particles will be larger than a naked coronavirus. It is therefore conceivable, but we believe unlikely, that the surgical helmet-hoods would efficiently filter coronavirus-containing particles. Second, we modified the PortaCount Plus by using disposable tubing rather than reusable tubing. As the disposable tubing and the tubing supplied by the manufacturer are both PVC, and of the same internal diameter and length, this change is unlikely to have made a difference in the results. Third, we only assessed the degree of respiratory protection provided by these devices. SARS is believed to be transmitted by contact of the virus with mucosal surfaces such as the eyes, as is the case with other respiratory viruses such as respiratory syncytial virus (*12*). Although both surgical helmet-hoods reduce the particle count compared to ambient counts, we believe this benefit may be counteracted by the fact that both devices direct a flow of gas into the eyes. Finally, the high particle count inside the hoods might have been due to the fan’s blowing particles off the hood material, the wearer’s head, or even the fan itself. In further experimentation, we found that when the surgical helmet was worn inside a PAPR system, the particle count inside the helmet was low, regardless of whether the fan was turned on or off (J. L. Derrick & C.D. Gomersall, unpub. data). It therefore seems unlikely that the particles are coming from any of these sources. Particles might also be drawn up from under the hood rather than through the hood material. In this case, the exact mechanism of entry would be irrelevant, as in both cases the indrawn air would be potentially contaminated if the patient had SARS.

Our data indicate that neither the Stryker T4 nor the Stackhouse FreedomAire helmet-hood filters enough particles of 0.02–1 μm in diameter to meet the standard for protective respirators. As the size of coronaviruses falls within this range, we recommend that neither device be used alone to protect against transmission of SARS.
